# Excoriation Disorder and Autism Spectrum Disorder: Clinical Challenges at the Intersection of Sensory Dysregulation and Repetitive Behaviors

**DOI:** 10.7759/cureus.117554

**Published:** 2026-10-06

**Authors:** Kayla K Leiber, Priya Pohani, Michelle Tashjian, Emily Garelick, Robert W Parker

**Affiliations:** 1 Dermatology, Alabama College of Osteopathic Medicine, Dothan, USA; 2 Dermatology, Nova Southeastern University, Davie, USA; 3 Dermatology, Philadelphia College of Osteopathic Medicine, Philadelphia, USA; 4 Pharmacology, Alabama College of Osteopathic Medicine, Dothan, USA

**Keywords:** autism spectrum disorder (asd), dermatillomania, repeated self-injurious behavior, sensory in autism and anxiety, skin excoriation

## Abstract

Excoriation disorder, also known as dermatillomania or skin-picking disorder, is characterized by recurrent, compulsive skin picking that leads to tissue damage, infection risk, pigmentary changes, and psychosocial impairment. While it is classified under the obsessive-compulsive and related disorders in the Diagnostic and Statistical Manual of Mental Disorders, Fifth Edition (DSM-5), research suggests a potential overlap with neurodevelopmental conditions, particularly autism spectrum disorder (ASD). In individuals with autism, skin-picking behaviors may be underrecognized or misattributed to atypical sensory processing or repetitive behaviors, creating significant diagnostic challenges. Because dermatologists are often the first to observe chronic excoriations, scarring, and infections, recognition of possible comorbidity with ASD is critical for timely referral and intervention. This literature review synthesizes current evidence on the overlap between excoriation disorder and ASD, including behavioral, sensory, and cutaneous contributors. It also highlights challenges in recognition and diagnosis, as skin-picking may be misattributed to autism itself and recognition is further complicated by communication barriers. Standard treatments such as cognitive behavioral therapy and pharmacologic agents often require autism-informed adaptation to account for sensory sensitivities, communication styles, and repetitive behavior profiles. Greater recognition of excoriation disorder within the context of ASD may help guide more effective, multidisciplinary care while ultimately improving patient outcomes.

## Introduction and background

Autism spectrum disorder (ASD) is a common neurodevelopmental condition, affecting an estimated one in 31 children [1] and one in 45 adults [2] in the United States. Among the many co-occurring conditions observed in individuals with autism, excoriation disorder (skin-picking disorder) is particularly relevant. ASD is defined in the Diagnostic and Statistical Manual of Mental Disorders, Fifth Edition (DSM-5) by persistent deficits in social communication and interaction together with restricted, repetitive patterns of behavior, interests, or activities, present in the early developmental period and causing clinically significant impairment [3]. It is termed a spectrum because presentations range widely, from individuals with intellectual disability and minimal language to verbally fluent individuals who require little support, and this heterogeneity shapes the form and severity of self-injurious behaviors, including skin picking. Excoriation disorder, also known as dermatillomania or skin-picking disorder, is characterized by recurrent, compulsive picking of the skin leading to tissue damage, infection risk, pigmentary changes, and psychosocial impairment. It is formally recognized in the DSM-5 as part of the category of obsessive-compulsive and related disorders, alongside trichotillomania, body dysmorphic disorder, and hoarding disorder. The condition is defined by recurrent skin picking that results in skin lesions, repeated attempts to decrease or stop the behavior, and significant distress or functional impairment. The behavior must not be attributable to the physiological effects of a substance (e.g., cocaine) or another medical condition (e.g., scabies), and it must not be better explained by another psychiatric disorder [3]. While symptoms often overlap with features of other psychiatric or developmental conditions, individuals with autism frequently meet the full criteria for excoriation disorder. This underscores that excoriation disorder represents a legitimate comorbid diagnosis in this population, rather than a behavior reducible to a symptom of autism. Recognizing it as distinct highlights an additional source of morbidity requiring focused clinical management and targeted research attention.

Repetitive and self-injurious behaviors such as skin picking represent a point of convergence across ASD and related neuropsychiatric conditions, contributing to its diagnostic complexity. Children with ASD, even in the absence of visible dermatitis, demonstrate increased transepidermal water loss, reduced stratum corneum hydration, and higher skin surface pH compared to neurotypical peers [4]. These epidermal abnormalities promote pruritus and tactile discomfort, which can act as direct triggers for skin-picking behavior. Clinically, individuals with autism may be underdiagnosed with excoriation disorder because skin-picking behaviors are often misattributed to autism itself rather than recognized as a comorbid condition [5]. Communication barriers can further complicate recognition. Most evidence-based treatments for skin-picking disorder were designed and tested in neurotypical populations. These interventions may be less effective or less accessible for autistic individuals, particularly those with sensory sensitivities or communication challenges. Clinicians therefore face a need to adapt existing approaches and develop autism-informed strategies for management.

Lifetime prevalence of excoriation disorder in the general population is estimated to be approximately 1-2%, with higher rates reported among individuals with co-occurring psychiatric conditions. Skin-picking behaviors are also disproportionately common among individuals with developmental disabilities [6]. Because excoriation disorder often presents with chronic excoriations, pigmentary changes, scarring, and recurrent infections, dermatologists are frequently the first clinicians to observe its physical manifestations. Early recognition in this setting is critical, as skin-picking injuries are often misattributed to primary dermatologic disease (e.g., eczema, bacterial infection) or accidental trauma. This diagnostic ambiguity is supported by clinical observations in children with ASD, where self-injurious behaviors frequently present with injury patterns localized to the legs, knees, and back, areas commonly associated with accidental trauma, making recognition of excoriation disorder particularly challenging [7]. Without considering excoriation disorder, clinicians may focus solely on wound care or antimicrobial therapy, missing the underlying behavioral driver.

In ASD, skin picking is frequently observed in the context of restricted and repetitive behaviors (RRBs) or as a form of self-injurious behavior. These behaviors may serve different functions from those typically seen in primary excoriation disorder, such as reducing sensory overload, providing sensory input, or regulating emotional states. The purpose of this review is to synthesize current findings on the co-occurrence of excoriation disorder and ASD. By examining current evidence and treatment approaches, this paper aims to clarify the diagnostic challenges and highlight the need for autism-informed interventions. Dermatologists play a critical role in recognizing when skin-picking may reflect excoriation disorder rather than solely a manifestation of autism-related behaviors. This distinction can guide appropriate referral, intervention, and collaboration with other specialists. Given the limited research base, the high prevalence, and the clinical consequences of under-recognition, there is a pressing need for further investigation into the intersection of excoriation disorder and ASD. The conceptual overlap between autism spectrum disorder and excoriation disorder, including shared sensory, behavioral, and dermatologic features, is illustrated in Figure 1.

**Figure 1:**
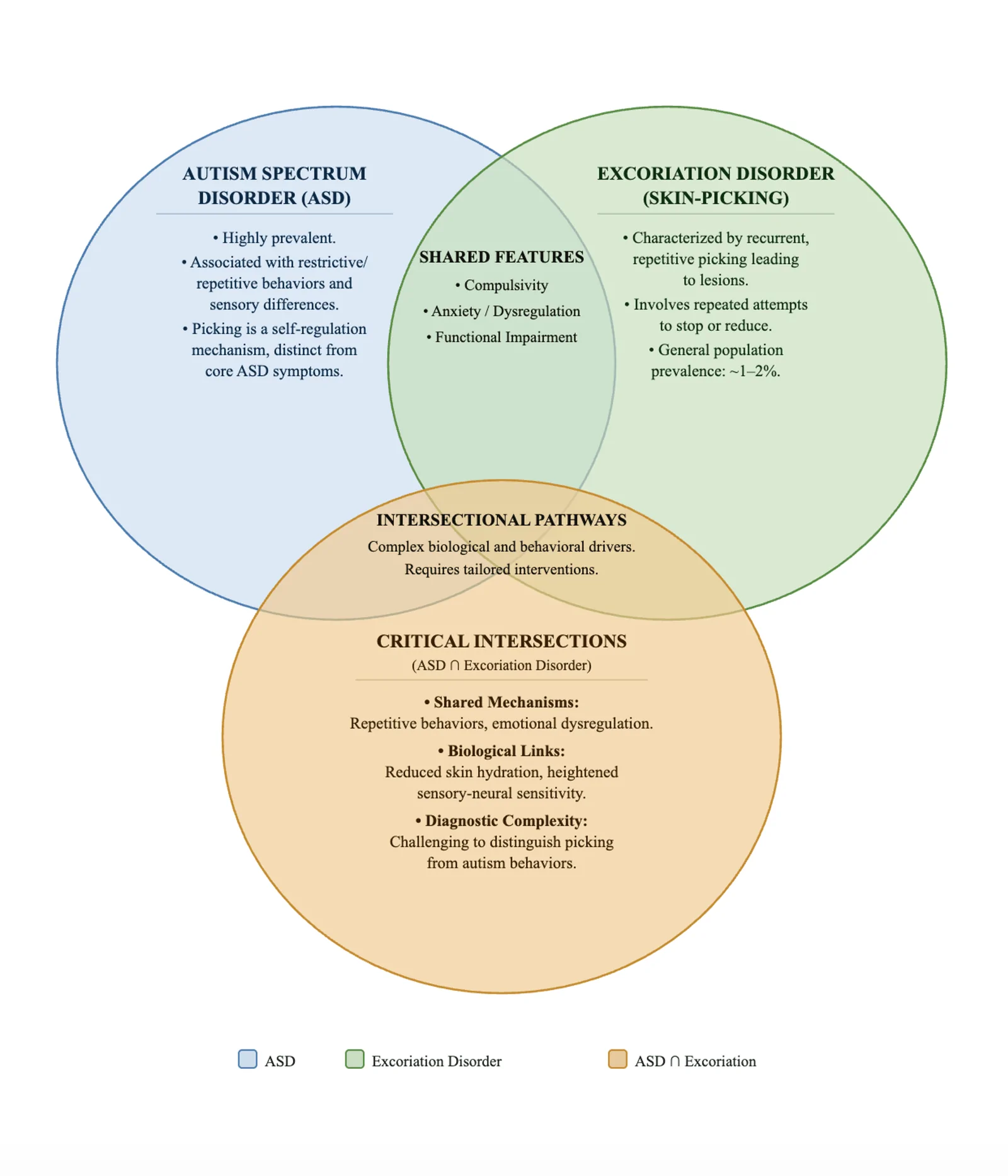
Conceptual overlap between autism spectrum disorder and excoriation disorder, highlighting shared sensory dysregulation, self-injurious behaviors, and dermatologic contributors The figure is an original conceptual schematic created by the authors using Canva.

## Review

Methods

This scoping review followed the Preferred Reporting Items for Systematic Reviews and Meta-Analyses Extension for Scoping Reviews (PRISMA-ScR) checklist [8]. A structured literature search was conducted across two electronic databases, PubMed and Google Scholar, covering the period from January 2015 to December 2025; searching and screening were carried out during the manuscript preparation window (searches last run in December 2025). All screening stages (title, abstract, and full-text review) were performed independently by two reviewers, with disagreements resolved by discussion and, when needed, adjudication by a third reviewer. The 2015 cutoff was selected to align the evidence base with the DSM-5 diagnostic framework [3], which formally recognizes excoriation disorder, and to capture the contemporary literature on ASD-skin comorbidity, sensory processing, and body-focused repetitive behaviors that has predominantly emerged since 2015.

The search combined controlled and free-text terms using Boolean operators. Synonymous terms within a concept block were joined with the operator OR, and distinct concept blocks were joined with the operator AND, structured as: (autism OR "autism spectrum disorder" OR ASD) AND (excoriation OR "skin-picking" OR dermatillomania OR "self-injury" OR "self-injurious behavior" OR "body-focused repetitive behavior"). Filters restricted results to English-language publications with full-text availability. The database-specific search terms and retrieved counts are summarized in Table 1.

**Table 1:** Publication search strategy

Search Engine	Search Terms	Publication Date	Language	Results Retrieved
PubMed	(autism) AND (excoriation) (autism) AND (skin-picking) (autism) AND (self-injury) (autism) AND (dermatillomania)	2015–2025	English	1,119
Google Scholar	(autism) AND (excoriation) (autism) AND (skin-picking) (autism) AND (skin) (autism) AND (dermatillomania)	2015–2025	English	384

Inclusion criteria were: (1) original research articles (observational, cross-sectional, cohort, longitudinal, experimental, clinical trials, or randomized controlled trials) or review articles; (2) publication in English between 2015 and 2025; (3) full-text availability; and (4) involvement of individuals with ASD together with discussion of excoriation disorder, skin-picking behaviors, or related self-injurious behaviors. Exclusion criteria were: (1) publications outside the 2015-2025 window (except foundational references retained selectively for historical or conceptual context); (2) non-English publications; (3) records without accessible full text; (4) articles that did not address both ASD and skin-picking/self-injurious behavior; and (5) case reports without generalizable findings, editorials, commentaries, and conference abstracts.

The final synthesis incorporated a range of article types, including original observational studies (cross-sectional, cohort, and longitudinal designs), experimental and clinical studies, randomized controlled trials, and narrative or systematic review articles, to reflect the heterogeneous and emerging nature of the evidence base, in which few ASD-specific excoriation disorder trials exist and much of the relevant knowledge is distributed across the dermatologic, neurobiologic, behavioral, and treatment literatures.

Consistent with PRISMA-ScR guidance, a formal risk-of-bias or critical-appraisal assessment of individual studies was not undertaken, as the aim was to map the breadth and nature of the available evidence rather than to grade the strength of effect estimates. The heterogeneity of included designs and the limited number of ASD-specific studies are addressed narratively in the Limitations.

The initial search yielded 1,503 records across both databases. Zotero (Corporation for Digital Scholarship, Vienna, VA) was used to manage references and remove duplicates using its automated duplicate-detection function, followed by manual verification. Following duplicate removal and exclusion of records identified as ineligible by automation tools, 895 records remained. These were screened by title independently by two reviewers, yielding 238 records for abstract review. Abstract screening narrowed this to 60 records eligible for full-text review, of which 19 studies met the inclusion criteria and were included in the final analysis. Foundational references published prior to 2015 were included selectively to provide historical or conceptual context. This strategy was designed to cast a broad, sensitive net across both a curated biomedical database (PubMed) and a wider indexing engine (Google Scholar), minimizing the risk of missing the sparse ASD-specific literature, while applying progressively stringent screening to retain only studies with direct relevance to the intersection of excoriation disorder and ASD, consistent with the PRISMA-ScR framework [8]. Extracted findings were synthesized narratively and grouped into four predefined thematic domains (dermatologic/skin-barrier, sensory/neurobiologic, behavioral/clinical, and treatment/intervention) by consensus among the reviewers; no quantitative pooling was performed given the heterogeneity of study designs. The selection process is summarized in Figure 2, and the characteristics and key findings of the 19 included studies, grouped by thematic domain, are presented in Table 2.

**Figure 2:**
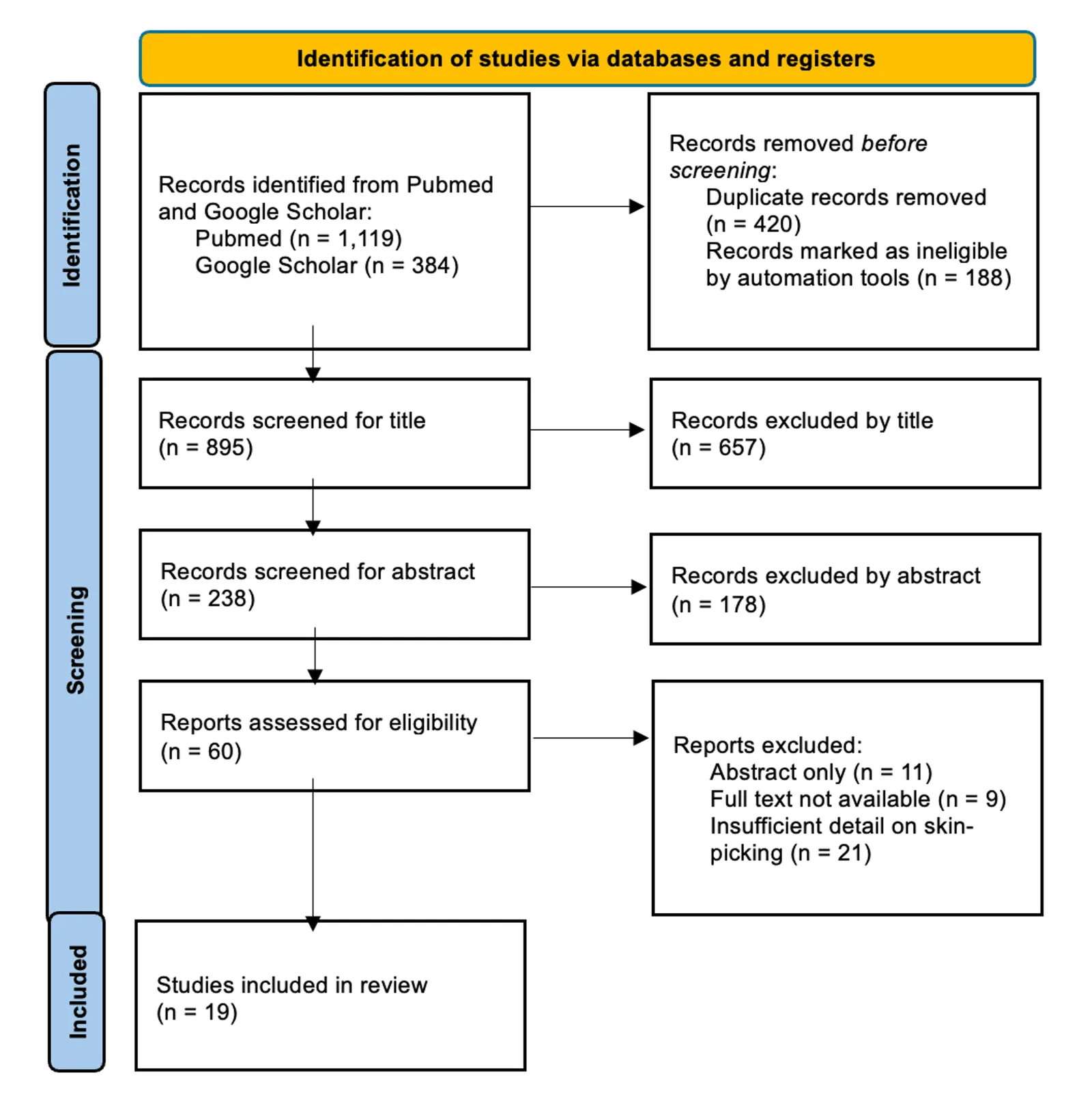
PRISMA flow diagram, figure made using the PRISMA 2020 template

**Table 2:** Summary of included studies (n = 19) ASD: autism spectrum disorder; fMRI: functional magnetic resonance imaging; SPD: skin picking disorder; BFRB: body-focused repetitive behavior; RCT: randomized controlled trial; CBT: cognitive behavioral therapy.

Category	Author	Study Type	Population	Key Findings
Dermatologic / Skin–Barrier	Wang et al. [4]	Observational	Children with ASD	Increased transepidermal water loss and altered skin barrier function.
	Al-Ayadhi et al. [9]	Cross-sectional	ASD vs controls	Higher prevalence of xerosis, pruritus, and skin disorders in ASD.
	Shin et al. [10]	Observational	Pediatric ASD	Overlap between atopic dermatitis and ASD suggests shared inflammatory pathways.
	Jameson et al. [11]	Cohort	Children with ASD	Atopic disease associated with increased ASD symptom severity.
	Man et al. [12]	Review	ASD populations	Proposed skin–brain axis linking inflammation and neurodevelopment.
Sensory / Neurobiologic	Bufo et al. [13]	Experimental	Children with ASD	Reduced autonomic response to affective touch.
	Capiotto et al. [14]	Experimental	Adults with ASD	Altered autonomic and hedonic tactile responses.
	Chien et al. [15]	Clinical study	Adults with ASD	Reduced intraepidermal nerve fiber density and altered sensory processing.
	Schienle et al. [16]	fMRI study	Skin-picking disorder	Abnormal brain activation during tactile processing.
	Collins et al. [17]	Review	SPD patients	Sensory dysregulation central to excoriation disorder.
Behavioral / Clinical	Laverty et al. [18]	Longitudinal cohort	Individuals with ASD	Self-injurious behavior persisted in approximately 44% over time.
	Slingsby et al. [7]	Observational	Children with ASD	Injury patterns often resemble accidental trauma, complicating recognition.
	Dempsey et al. [19]	Observational	Children/adolescents with ASD	High prevalence of self-injurious behaviors.
	Rojahn et al. [20]	Longitudinal	Developmental delay	Early stereotyped behaviors predict later self-injury.
Treatment / Intervention	Asplund et al. [21]	Clinical trial	BFRB patients	Internet-based therapy reduced skin-picking symptoms.
	Gallinat et al. [22]	RCT	Skin-picking disorder	CBT-based self-help significantly reduced symptom severity.
	Murase et al. [23]	Review	BFRB populations	Digital interventions effective but depend on engagement.
	Lochner et al. [24]	Systematic review	Skin-picking disorder	Habit reversal training and CBT are most effective.
	Grant et al. [25]	Review	BFRB disorders	Updated evidence on pharmacologic and behavioral treatments.

Discussion

Diagnostic Challenges and Clinical Consequences in ASD

The diagnostic overlap between excoriation disorder and ASD is substantial because both conditions feature repetitive, body-directed behaviors, yet the two arise from partly distinct mechanistic drivers. In excoriation disorder, picking is typically driven by urges, tension reduction, and impaired inhibitory control [26], whereas in ASD the same behavior may function as sensory-seeking, sensory-avoidant, self-regulatory, or communicative self-injury [5]. This shared surface phenotype but divergent function is the central reason the disorders are difficult to disentangle at the bedside.

Diagnosis and treatment planning can be especially challenging in autistic individuals with limited verbal abilities. Nonverbal IQ (NVIQ) is associated with restricted and repetitive behaviors (RRBs) in older children with autism [27], suggesting that those with lower NVIQ may be more vulnerable to excoriation-type behaviors. At the same time, communication barriers in this group can obscure distress, motivation to change, or insight into the behavior, making it difficult for clinicians to determine whether diagnostic criteria for excoriation disorder are met. This combination highlights the importance of tailored assessment tools and autism-informed therapeutic approaches. A key contributor to under-recognition is diagnostic overshadowing, in which clinicians attribute skin-picking to autism itself rather than identifying it as a co-occurring disorder, a bias particularly consequential in minimally verbal patients who cannot report the distress, guilt, or urge-to-stop central to the excoriation-disorder criteria [28].

Assessment challenges therefore require deliberate adaptation. Validated instruments such as the skin picking scale can structure screening, but self-report tools assume a level of insight and verbal report that may not apply in ASD. Incorporating caregiver-reported behavioral observation, functional behavioral assessment, and direct inspection of lesion distribution can supplement self-report and reduce reliance on subjective distress as the sole diagnostic anchor. Longitudinal data further indicate that self-injurious behaviors, including skin picking, persist in a substantial proportion of individuals with ASD over time, with studies demonstrating persistence rates approaching 44% [18].

Failure to correctly identify excoriation disorder can have significant clinical and psychosocial consequences, including repeated infections, delayed wound healing, and permanent scarring, with downstream effects on self-esteem and social functioning [26]. Conversely, overdiagnosing excoriation disorder without recognizing underlying autism is also problematic: treatment may focus exclusively on habit reversal or pharmacotherapy without addressing the sensory sensitivities, communication barriers, or RRB patterns that sustain the behavior, leading to ineffective treatment and unnecessary polypharmacy [29]. Misdiagnosis also has implications for psychiatric comorbidities; untreated excoriation disorder has been associated with higher rates of anxiety and depression, while unrecognized autism can leave patients without tailored supports [30]. Dermatologists and psychiatrists are therefore encouraged to maintain a high index of suspicion and to collaborate in multidisciplinary settings. Real-world treatment patterns also suggest a higher dermatologic burden in ASD, underscoring the need for clear, sensory-tolerant skin-care plans [12].

Neurobiological Considerations

Central circuitry: Excoriation disorder is increasingly understood as a disorder of impaired inhibition and reward dysregulation, with overlapping mechanisms observed in both obsessive-compulsive spectrum conditions and autism spectrum disorder (ASD). Functional neuroimaging and cognitive testing implicate dysfunction in the cortico-striato-thalamo-cortical (CSTC) circuits, particularly involving the anterior cingulate cortex, orbitofrontal cortex, and basal ganglia. These networks regulate habit formation and motor control, and their dysregulation has been demonstrated directly in excoriation disorder [31]. Because excoriation disorder is classified within the same obsessive-compulsive spectrum, and because ASD has also been linked to CSTC abnormalities and difficulties with inhibitory control, it is plausible that similar circuit dysfunction contributes to repetitive grooming and skin-picking behaviors in autistic individuals; dorsal-striatal functional connectivity has been directly linked to repetitive-behavior dimensions in neurodevelopmental disorders, supporting a shared striatal substrate [32].

Peripheral and sensory mechanisms: Complementing these central mechanisms, genetic data suggest that disturbances of peripheral skin sensation may also contribute causally to ASD pathogenesis, reinforcing the role of sensory dysregulation as a mechanistic bridge to body-focused repetitive behaviors like excoriation disorder [33]. Human biopsy studies further support this link, showing reduced intraepidermal nerve fiber density in autistic adults, associated with altered thermal detection, atypical nociceptive processing, and autism symptom severity [15]. In addition to cutaneous abnormalities, atypical sensory processing of touch has been documented in ASD. For example, children with autism show reduced autonomic responses (pupil dilation, skin conductance, heart rate changes) to gentle stroking across both C-tactile (CT) fiber-rich and CT-poor skin areas compared to typically developing peers. This pattern suggests autonomic dysregulation and reduced tactile responsivity, which may contribute to altered arousal regulation and reinforce repetitive skin-focused behaviors [13]. Recent work on affective touch adds further support to this sensory-autonomic dysregulation model. Studies of both children [13] and adults [14] with ASD demonstrate blunted autonomic nervous system responses, including reduced skin conductance and absent differentiation between affective versus neutral touch, despite self-reported hedonic ratings being preserved or even heightened. This dissociation between subjective and physiologic responses suggests impaired integration of sensory input and autonomic regulation, a mechanism that may similarly underlie maladaptive repetitive behaviors such as skin-picking.

Neurotransmitter systems: Parallel findings in skin-picking disorder reinforce this model: patients demonstrate aberrant parietal and frontal activation during caress-like touch, and report reduced pleasantness but heightened arousal and urge to pick, indicating dysfunctional attentional and affective touch processing [16]. Neurotransmitter systems also appear to play a critical role. Dysregulation of dopamine in the mesolimbic reward pathway may reinforce the immediate sensory or emotional relief gained from skin picking, while serotonin imbalance may impair behavioral inhibition. This dual pathway model helps explain why both selective serotonin reuptake inhibitors (SSRIs) and dopamine-modulating agents such as risperidone have shown efficacy in some individuals [25]. Evidence remains limited; randomized ASD-specific excoriation disorder trials are needed. It should also be emphasized that much of this mechanistic model is inferential: the circuit- and neurotransmitter-level findings derive largely from animal models, cross-sectional imaging, and analogy to obsessive-compulsive disorder (OCD), and cannot be tested through the controlled interventional manipulations available in preclinical work, so these pathways remain hypothesized rather than established in autistic individuals.

Relationship to the obsessive-compulsive spectrum: A conceptual question that this review does not fully resolve is whether excoriation disorder is more accurately understood as an obsessive-compulsive spectrum condition than as an ASD-related behavior. In DSM-5, excoriation disorder is classified among the obsessive-compulsive and related disorders alongside trichotillomania and body dysmorphic disorder, and its neurocircuitry, particularly CSTC dysfunction and impaired inhibitory control, is shared substantially with OCD rather than being specific to autism. Under this interpretation, the co-occurrence of skin-picking with ASD may partly reflect the elevated general burden of obsessive-compulsive spectrum and body-focused repetitive behaviors in autistic individuals, rather than a mechanism unique to autism itself. This is an important competing view: some evidence frames excoriation disorder primarily as an OCD-related condition whose association with ASD is mediated by shared repetitive-behavior and inhibitory-control phenotypes, whereas the sensory and dermatologic mechanisms emphasized elsewhere in this review suggest additional, autism-specific contributions. These perspectives are not mutually exclusive, and disentangling them will require studies that directly compare excoriation disorder, OCD, and ASD within the same design. Acknowledging this tension is important to avoid overstating a direct or autism-specific causal link.

Dermatologist’s Role in Care

Children with ASD show abnormal skin barrier function, including increased transepidermal water loss and reduced hydration, which can intensify pruritus and discomfort. These physiologic changes may increase the likelihood of skin-picking behaviors and should be considered in diagnostic evaluations [4]. Beyond barrier dysfunction, emerging research shows that inflammatory dermatoses such as severe atopic dermatitis and psoriasis are disproportionately represented in individuals with ASD, and maternal immune activation involving IL-17A pathways has been linked to autism risk in offspring. This overlap is further supported by clinical studies demonstrating phenotypic and inflammatory similarities between atopic dermatitis and autism spectrum disorder [10]. Together, barrier dysfunction and inflammation may create a biologic vulnerability to autism traits and body-focused repetitive behaviors such as excoriation [12]. Genetic and epigenetic studies further reinforce this overlap, with copy number variants, filaggrin mutations, and micro ribonucleic acid (microRNAs) such as miR-146a and miR-155 implicated in both ASD and atopic dermatitis, highlighting shared immune-cutaneous pathways that may predispose to excoriation behaviors [34].

Dermatologists are often the first to identify problematic skin-picking. Their role extends beyond wound care to include screening, protective strategies, and timely referral to psychiatry. Dermatologists also play a key role in initiating multidisciplinary care pathways, particularly for patients with underlying psychiatric comorbidities.

Beyond wound care, dermatologists are well positioned to initiate the barrier-repair and sensory-tolerant skin-care regimens detailed in the treatment approaches section, which may modulate the pruritus and tactile triggers that reinforce repetitive behaviors and thereby represent a therapeutic application of the skin-brain connection.

Treatment Approaches

Practical management of co-occurring excoriation disorder and ASD is best conceptualized as a multimodal, autism-informed strategy integrating behavioral, pharmacologic, sensory, and dermatologic components.

Behavioral interventions: Cognitive behavioral therapy (CBT) and habit reversal training (HRT) remain the gold standard for treating excoriation disorder. These approaches focus on increasing awareness of triggers, building competing responses, and reinforcing adaptive coping strategies. However, in autistic individuals, modifications may be required to account for communication barriers, cognitive profiles, or sensory sensitivities. For example, visual aids, caregiver involvement, and structured reinforcement systems may improve accessibility and effectiveness. In addition, competing stimulus assessments (CSA) have been shown to help identify items that reduce automatically maintained skin picking in autistic children, with noncontingent access to a preferred stimulus demonstrating durable reductions in behavior, underscoring the value of individualized behavioral tools [35].

Pharmacologic strategies: N-acetylcysteine (NAC), a glutamatergic modulator, has shown efficacy in excoriation disorder, with 47% of treated adults showing significant improvement compared to 19% on placebo [36]. Notably, NAC appears to primarily reduce urges to pick rather than automatic picking behaviors, which may limit its effectiveness in autistic individuals who pick with reduced conscious awareness. In ASD specifically, meta-analytic evidence demonstrates that NAC reduces irritability and hyperactivity and improves social awareness, though direct effects on skin-picking behaviors in this population require further study [37,38].

Adjunctive and sensory-friendly strategies: Adjunctive measures may be especially valuable in ASD, where sensory regulation plays a central role. These include the use of barrier creams, hydrocolloid dressings, protective gloves, soft cotton fabrics, and frequent emollient application to minimize tactile triggers and reduce reinforcement of the picking behavior. Digital treatments also show promise. In a psychiatric clinic setting, a 10-week therapist-guided online program that combined habit reversal with acceptance-based techniques was found to be feasible, acceptable, and effective, with large reductions in symptoms that lasted at follow-up [22]. Similarly, the SaveMySkin randomized trial showed that a 12-week CBT-based self-help program led to significant reductions in skin-picking severity compared to waitlist controls, and demand for participation was very high [23]. A recent review of 12 randomized trials concluded that internet-based self-help options such as self-guided habit reversal and decoupling techniques can reliably reduce symptoms and improve quality of life. The “Free from BFRB (body-focused repetitive behaviors)” website was the most studied and well-rated resource, although therapist-guided versions still tend to produce better outcomes, and patient engagement is crucial [24]. These findings align with systematic reviews, which consistently emphasize behavioral therapy, particularly habit reversal training (HRT) and acceptance-enhanced CBT, as the most effective nonpharmacologic strategies for excoriation disorder, even though trial data remain limited [25]. While SSRIs and glutamatergic agents such as N-acetylcysteine may play a role, behavioral strategies remain the cornerstone of care [24]. Where dry, itchy skin contributes to picking, sensory-tolerant skin care should be treated as mechanistic rather than cosmetic (see Barrier-repair therapy below). Self-help behavioral techniques show promise for increasing treatment accessibility. Habit replacement, a variant of HRT that incorporates self-soothing through circling nonharmful movements, demonstrated significant reductions in BFRBs compared to waitlist controls. Comparative trials suggest that concurrent application of multiple techniques (HRT, decoupling, decoupling in sensu) may produce additive effects, though completion rates and subjective acceptability vary across approaches.

Barrier-repair therapy: Barrier-repair therapy has recently emerged as a promising adjunct. Because epidermal barrier dysfunction in ASD (increased transepidermal water loss, reduced hydration) can drive the pruritus and tactile discomfort that reinforce picking, a stepwise regimen of ceramide-dominant emollients, pH-balanced cleansers, hydrocolloid dressings on common picking sites, and fragrance-free cleansers is recommended as mechanistic care rather than a cosmetic adjunct. Small studies suggest such regimens improve both skin integrity and autism-related symptom burden, supporting the skin-brain connection as a therapeutic target.

Research Gaps and Future Directions

Further studies are needed to clarify the neurobiological components of excoriation disorder in ASD, particularly regarding dopamine and serotonin signaling in reward pathways. Emerging genetic evidence indicates that skin sensation disturbances may serve as a quantifiable biomarker of ASD [33], raising the possibility that early identification of tactile hypersensitivity could help stratify subgroups at elevated risk for excoriation disorder or related body-focused repetitive behaviors. Longitudinal, controlled trials could help determine whether multimodal approaches (behavioral plus pharmacologic) are more effective than pharmacotherapy alone.

Environmental triggers, sensory sensitivities, and caregiver involvement should be systematically studied. Future studies should also clarify developmental trajectories, as stereotyped behaviors in infancy have been shown to precede later self-injury [20]. Identifying such early markers could inform preventive interventions before excoriation disorder becomes present. There is also a need for clinician training modules to improve early recognition and multidisciplinary management across dermatology, psychiatry, and primary care. Multiregional, clinically verified cohorts are needed to generalize beyond the single-region and to link barrier and trigger reduction to objective picking outcomes [12].

Specifically needed are, (1) prospective cohort studies tracking skin-picking behaviors from early childhood through adolescence in autistic populations to identify developmental trajectories and early markers; (2) randomized controlled trials of autism-adapted HRT versus standard HRT versus pharmacotherapy; (3)component analysis and controlled trials of multimodal interventions determining which elements (behavioral, pharmacologic, barrier-repair) contribute most to outcomes, integrating objective skin metrics with behavioral endpoints to test whether improving epidermal health reduces excoriation severity in ASD; and (4) biomarker studies integrating genetic markers of skin sensation disturbance with clinical dermatologic phenotypes to enable precision-medicine approaches to risk stratification and early intervention.

This review has several limitations. Restricting the search to two databases (PubMed and Google Scholar), to English-language publications, and to records with accessible full text may have missed relevant studies and could introduce geographic, cultural, and indexing bias. Heterogeneous definitions of skin-picking versus general self-injurious behavior across studies limit precise prevalence estimates. Most importantly, the paucity of ASD-specific excoriation disorder trials means many treatment recommendations are extrapolated from neurotypical populations or general ASD self-injury literature. Future research should address these gaps through standardized assessment tools and autism-specific treatment trials.

Future research should consider how findings on excoriation disorder in ASD may generalize to other body-focused repetitive behaviors (BFRBs) such as trichotillomania (hair pulling) and onychophagia (nail biting). These disorders share overlapping neurobiological pathways, including CSTC circuit dysfunction and serotonergic-dopaminergic imbalance, as well as common behavioral drivers such as tension reduction and sensory reinforcement [39]. Given their frequent co-occurrence, studying these behaviors together could clarify whether autism-specific adaptations in diagnosis and treatment are broadly applicable across the BFRB spectrum. Comparative designs similar to recent work contrasting OCD, trichotillomania, and controls [40] could be extended to excoriation disorder, helping to distinguish shared from disorder-specific mechanisms and guiding autism-informed therapeutic strategies. This comparative evidence suggests that excoriation disorder may similarly reflect elevated autistic traits and highlights the need for autism-informed diagnostic and therapeutic approaches across the BFRB spectrum, with comparative trials examining excoriation disorder and trichotillomania in autistic populations helping to identify shared versus distinct mechanisms.

## Conclusions

Excoriation disorder in autistic individuals represents a distinct, frequently under-recognized comorbidity whose diagnosis is complicated by symptom overlap with restricted and repetitive behaviors, communication barriers, and variable insight, and whose relationship to ASD is bidirectional-autistic traits can predispose to skin-picking that meets criteria for excoriation disorder, while persistent excoriation may itself prompt first-time evaluation for autism; because dermatologists are often the first to observe chronic excoriations, scarring, and recurrent infection, they play a pivotal role in maintaining a low screening threshold using validated tools such as the skin picking scale, inquiring about autism diagnosis or traits, assessing sensory sensitivities and communication barriers, and initiating collaborative, multidisciplinary care pathways with psychiatry and developmental specialists, while shared sensory, autonomic, and cortico-striato-thalamo-cortical mechanisms link the two conditions and support multimodal, autism-informed management that integrates modified behavioral therapy, judicious pharmacotherapy, sensory-friendly adjuncts, and barrier-repair strategies that may serve dual diagnostic and therapeutic function, together underscoring that greater clinician awareness, specialized training, and dedicated ASD-specific research are essential to avoid both underdiagnosis and misattribution and to improve outcomes for this vulnerable population.
